# Phylogenetic endemism of the orchids of Megamexico reveals complementary areas for conservation

**DOI:** 10.1016/j.pld.2022.03.004

**Published:** 2022-03-25

**Authors:** Brandon E. Gutiérrez-Rodríguez, Marilyn Vásquez-Cruz, Victoria Sosa

**Affiliations:** a Instituto de Ecología AC, Carretera Antigua a Coatepec 351, El Haya, 91073 Xalapa, Veracruz, Mexico; b Unidad de Genómica Avanzada, Laboratorio Nacional de Genómica para La Biodiversidad, Centro de Investigación y Estudios Avanzados Irapuato, Libramiento Norte Carretera León Km. 9.6, 36821 Irapuato, Guanajuato, Mexico

**Keywords:** Epiphytes, Geophytes, Orchids, Phylogenetic endemism, Weighted endemism

## Abstract

Orchid diversity provides a unique opportunity to further our understanding of biotic and abiotic factors linked to patterns of richness, endemism, and phylogenetic endemism in many regions. However, orchid diversity is consistently threatened by illegal trade and habitat transformation. Here, we identified areas critical for orchid conservation in the biogeographic province of Megamexico. For this purpose, we evaluated orchid endemism, phylogenetic diversity, and phylogenetic endemism within Megamexico and characterized orchid life forms. Our results indicate that the majority of the regions with the highest estimates of endemism and phylogenetic endemism are in southern Mexico and northern Central America, mostly located on the Pacific side of Megamexico. Among the most important orchid lineages, several belong to epiphytic lineages such as Pleurothallidinae, Laeliinae and Oncidiinae. We also found that species from diverse and distantly related lineages converge in montane forests where suitable substrates for epiphytes abound. Furthermore, the southernmost areas of phylogenetic diversity and endemism of Megamexico are in unprotected areas. Thus, we conclude that the most critical areas for orchid conservation in Megamexico are located in southern Mexico and northern Central America. We recommend that these areas should be given priority by the Mexican system of natural protected areas as complementary conservation areas.

## Highlights

• Endemism and phylogenetic endemism were used to identify priority areas for conservation of orchids native to Megamexico.
• Analyses of life form prioritized conservation of areas along the Pacific edge of Megamexico for epiphytes.
• Highest estimations of endemism and phylogenetic endemism are in mountains in southernmost Megamexico in non-protected areas.
• Areas of endemism that harbor evergreen and cloud forests with tall dominant trees offer favorable substrates for epiphytes.

## Graphical abstract

**Figure:**
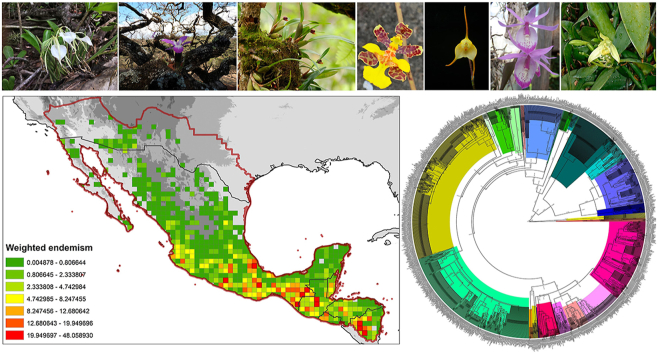

## 1 Introduction

Orchidaceae constitute one of the most diverse angiosperm families, as a number of lineages have undergone an accelerated net rate of diversification (Givnish et al., 2015). Accordingly, research has focused on understanding the biotic and abiotic factors that underlie patterns of orchid richness and endemism in many biogeographic regions of the world (Ackerman et al., 2007; Phillips et al., 2010; Acharya et al., 2011; Tsiftsis et al., 2011; Zhang et al., 2017; Garsuta Betanio and Buenavista, 2018; Crain and Ferná ndez, 2020; Mehrabian et al., 2021). Diversification of orchids has been associated the evolution of pollinia, epiphytic life form, CAM photosynthesis, pollination by Lepidoptera butterflies and moths or by Euglossine bees, and tropical distribution in mountain systems (Chase et al., 2015; Givnish et al., 2015). In the Neotropics, rapid speciation in lineages such as Pleurothallidinae, Maxillariinae and Cymbidieae has been linked to mountain uplift across all elevation zones (Kirby, 2016; Pérez–Escobar et al., 2017).

Advanced measures of biodiversity such as endemism, phylogenetic diversity and phylogenetic endemism have been used to identify and understand the ecological, evolutionary, and biogeographic processes that have shaped the evolutionary history of a region (Costion et al., 2015; Thornhill et al., 2016; Simón–Porcar et al., 2018). These measures can also be used to identify priority areas and/or lineages for conservation (Faith, 1992; Faith et al., 2004; Rosauer et al., 2009; González–Orozco et al., 2015; Laity et al., 2015).

Megamexico forms part of a region that is considered a hotspot of biodiversity (Myers et al., 2000; Castillo–Pérez et al., 2019). The orchids of Megamexico — many of which are endemic — represent a significant element of the plant diversity of the countries comprising the Mesoamerican hotspot (Ames and Correll, 1985; Dressler, 1993; Hágsater et al., 2005). However, many species of this plant family are endangered due to illegal trade and habitat destruction (Flores–Palacios and Valencia–Díaz, 2007; Jiménez–López et al., 2019). Furthermore, many orchid species figure prominently on lists of the threatened species of this region (e.g., Hágsater and Soto–Arenas, 1998; Kull et al., 2006). Protected natural areas in Megamexico are located in a variety of habitats and regions; however, they are scarce in mountain areas where epiphytic orchids have been recorded as abundant and where habitat destruction is accelerating. Thus, it is crucial to determine the areas with the most endemics in order to propose conservation strategies (Soto–Arenas et al., 2007; Crain and Tremblay, 2014).

One approach to understanding the ecological and evolutionary processes that drive endemism and phylogenetic endemism of particular lineages or of entire plant communities relies on examining functional attributes (see Keppel et al., 2018). For orchids, complex patterns of diversity and endemism in islands around the world have been associated with life form (Taylor et al., 2021). Research has suggested that the elevated endemism of orchids in tropical Andean forests has been driven by rapid speciation processes in epiphytic orchids (Küper et al., 2004). Furthermore, the threat of extinction varies as a function of life form in orchids. Epiphytes are more sensitive to habitat destruction because they depend on arboreal vegetation (Rasmussen and Rasmussen, 2018; Gale et al., 2018), while terrestrial and mycoheterotrophic orchids depend on their association with fungi (Swarts and Dixon, 2009; Sosa et al., 2018). Thus, if life form is considered when estimating advanced measures of biodiversity for lineages or species, it is possible to determine differences in threat levels to this functional character in specific locations.

In this study, we aimed to understand the evolutionary history of the native orchids in the biogeographic province of Megamexico and identify areas crucial for conservation. For this purpose, we (1) determined areas of endemism, phylogenetic diversity, and phylogenetic endemism for the orchids of Megamexico, (2) compared life forms in these areas, and (3) identified priority areas for their conservation.

## 2 Materials and methods

### 2.1 Study area

Rzedowski (1991) used affinities and endemics listed in the Mexican phanerogamic flora to delimit a biogeographic region named Megamexico, which ranges from the deserts of northern Mexico (e.g., Sonoran Desert, Chihuahuan Desert, and the deserts of Tamaulipas) to the northern regions of Central America. This biogeographic region comprises varied ecosystems: evergreen tropical forests, sub-deciduous tropical forests, seasonally tropical deciduous forests, thorn forests, xerophilous scrub, grasslands, *Quercus* forests, coniferous forests, cloud forests, and aquatic and underwater vegetation (Rzedowski, 1991). The elevation of Megamexico ranges from sea level to approximately 4500 m a.s.l. in the highest mountains of the Trans-Mexican Volcanic Belt (Rzedowski, 1991). In addition, the southernmost region of Megamexico is characterized by converging mountain systems, including the Sierra Madre del Sur, the Chiapas Highlands, the Sierra Madre de Guatemala, and other minor sierras in Central America, such as the Sierra de los Cuchumatanes, the Sierra de las Minas, and the Sierra Agalta (Ferrusquía–Villafranca, 1993). These converging mountain systems create complex topographies.

### 2.2 Checklist of species and their life form

We based our checklist of native orchids in Megamexico on the literature, published flora, and monographs (Appendix A). Classification followed Chase et al. (2015) (subfamily, tribe, subtribe). Life form was noted for every species: terrestrial (geophyte/mycoheterotrophic), epiphyte (deciduous/perennial), and rupicolous (over rocks). These characters were encoded as follows: 1 was assigned if the species in question had a given functional attribute and 0 if it did not (Appendix B). Life form followed the classification by Raunkiær (1934).

### 2.3 Database

We checked our newly constructed checklist against the electronic database of Global Biodiversity Information Facility (GBIF.org, 2020; https://doi.org/10.15468/dl.ypf5sk). Georeferences were carefully reviewed to avoid any duplication. The database was complemented by personal examination of specimens in the main Mexican herbaria (AMO, ENCB, IBUG, IEB, MEXU, XAL). The acronyms follow Thiers (2017). Records lacking latitude, longitude, and elevation data were georeferenced with the help of ArcView GIS v.3.3 (ESRI, 2002) and Google Earth (Google, 2007). Records with imprecise locality information were excluded. Georeferences outside Megamexico were recorded as well for the species whose ranges extend beyond this region.

### 2.4 Phylogenetic analyses

To estimate phylogenetic diversity and phylogenetic endemism, phylogenetic relationships were identified using the methodology proposed by Jin and Qian (2019) with the V.PhyloMaker package in R 3.4.0 (R Development Core Team, 2018). This method is based on the megaphylogeny of seed plants by Smith and Brown (2018) and Zanne et al. (2014) for terrestrial plants. The mega-tree used by V.PhyloMaker is a combination of both phylogenies with updates and fixes. To be considered in phylogenetic analyses, species can be added by this software in three ways: 1) species are added as basal polytomies within their respective genera; 2) genera or species are added randomly within their families or genera; 3) genera or species are added to their families as polytomies at the midpoint of the genus branch length. Following the recommendations of Allen et al. (2019), Mishler et al. (2020) and Qian and Jin (2021), we used the last strategy to construct the orchid phylogeny of Megamexico (Appendix C). In so doing, the species on our checklist that lacked sequences in GenBank were added as polytomies at the midpoint of the branch of the genus to which each belongs. Terminals were added with zero length to retain the ultrametricity of the tree.

### 2.5 Species richness, weighted endemism, phylogenetic diversity, and phylogenetic endemism

Species richness, weighted endemism, phylogenetic diversity, and phylogenetic endemism were estimated using Biodiverse v.3.1 (Laffan et al., 2014). We used a grid size of 0.5° × 0.5° to allow comparisons of our results with those of previous studies (Sanginés–Franco et al., 2015; Cuéllar–Martínez and Sosa, 2016; Sosa et al., 2018).

Species richness was calculated by computing species present in each grid and giving each a weight of 1. Weighted endemism was calculated following Linder (2001) and Laffan et al. (2016). Weighted endemism is a rank-weighted richness score. Thus, the value of each species present in the grid is weighted so that it is proportional to the fraction of its distribution range in all grids. To determine the value of a cell, the resulting values of all the species that were present in that cell were added (Crisp et al., 2001; Linder, 2001; Laffan et al., 2016). Weighted endemism prioritizes areas with a greater number of species with restricted ranges of distribution (Crisp et al., 2001).

Phylogenetic diversity was calculated as the sum of the branch lengths of a phylogenetic tree linking a set of terminal taxa to the root of the tree, as a proportion of the total length of the tree (Faith, 1992; Faith et al., 2004; Allen et al., 2019). In phylogenetic diversity, the ranges of all branches of the tree connecting taxa are incorporated and not just the terminal branches (Rosauer et al., 2009). Phylogenetic diversity accounts for the extent of the distribution range of the taxa that are present in an area along with all the taxa in the phylogeny, regardless of their distribution range. Also, phylogenetic diversity considers not only the rank of each taxon, but also that of each branch. The result is the total of the branch length within the clade range for each branch in the connecting path linking a group of taxa to the root of the tree (Rosauer et al., 2009). To identify grid cells with important values for phylogenetic diversity and phylogenetic endemism, 1000 randomizations were made in Biodiverse v.3.1. The most significant estimates are displayed on the maps.

We also measured the ranges of non-endemic species and graphed them in R v.3.1. (R Development Core Team, 2018) to understand how they affect estimations of weighted endemism, phylogenetic diversity, and phylogenetic endemism. We graphically compared the frequency distribution of life forms (geophytes, mycoheterotrophs, perennial, deciduous, and rock-dwelling epiphytes) to phylogenetic diversity and corrected weighted endemism values (see Appendix D). Corrected weighted endemism corrects for the species richness effect by measuring the percentage of endemics in a grid cell (Crisp et al., 2001). Results of phylogenetic diversity and corrected weighted endemism obtained by Biodiverse v.3.1 were exported in CSV format to be processed and graphed in R v.3.4.0. (R Development Core Team, 2018).

In addition, grids of phylogenetic diversity and phylogenetic endemism were superimposed on a shapefile of the Natural Protected Areas System of Mexico (http://sig.conanp.gob.mx/website/pagsig/listanp/) to determine whether the cells with the highest values were located in a protected area.

## 3 Results

### 3.1 Species and life forms

**Fig. 1:**
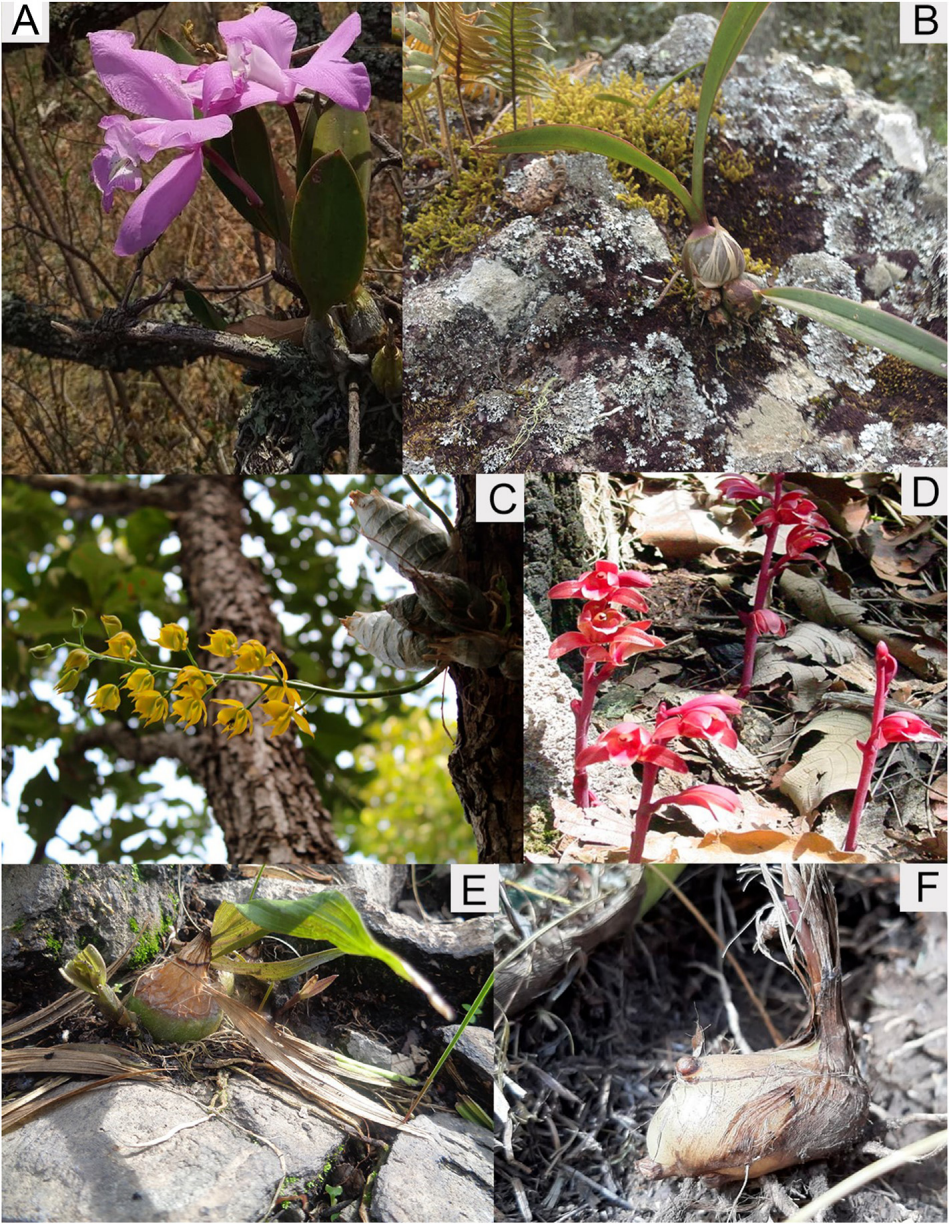
Life forms of the orchids of Megamexico. **A.** Perennial epiphyte (*Laelia speciosa*). **B.** Rupicolous orchid (*Encyclia* sp.). **C.** Deciduous epiphytes (*Mormodes* sp.). **D.** Mycoheterotrophic orchid (*Hexalectris spicata*). **E.** Geophyte displaying corm (*Bletia punctata*). **F.** Geophyte displaying the corm, the perennating organ that grows under the surface (*Bletia parkinsonii*).

A total of 47,407 georeferenced records of Orchidaceae species in Megamexico were analyzed. This number corresponds to 1,732 species, which in turn are grouped into 189 genera, 36 subtribes and 16 tribes. There are representatives from four of the five subfamilies of Orchidaceae: Vanilloideae, Cypripedioideae, Orchidoideae, and Epidendroideae. Of the total number of genera present, 18 are highly diverse and together comprise 951 species (54.6% of the total). The majority of Orchidaceae species records are epiphytes (1,224), of which 1,153 are plants with evergreen leaves and 71 are species with deciduous leaves. One hundred and four species are rupicolous. Finally, 505 are terrestrial, of which 23 are mycoheterotrophic (Fig. 1).

### 3.2 Phylogenetic analyses

The phylogeny of the 1,732 species found in Megamexico (Appendix C) retrieved monophyletic groups at the subtribe level. Following the approach of Jin and Qian (2019), the 312 species that lacked the molecular markers utilized by Zanne et al. (2014) were added to the phylogeny (see Materials and Methods).

### 3.3 Endemism and phylogenetic endemism

**Fig. 2:**
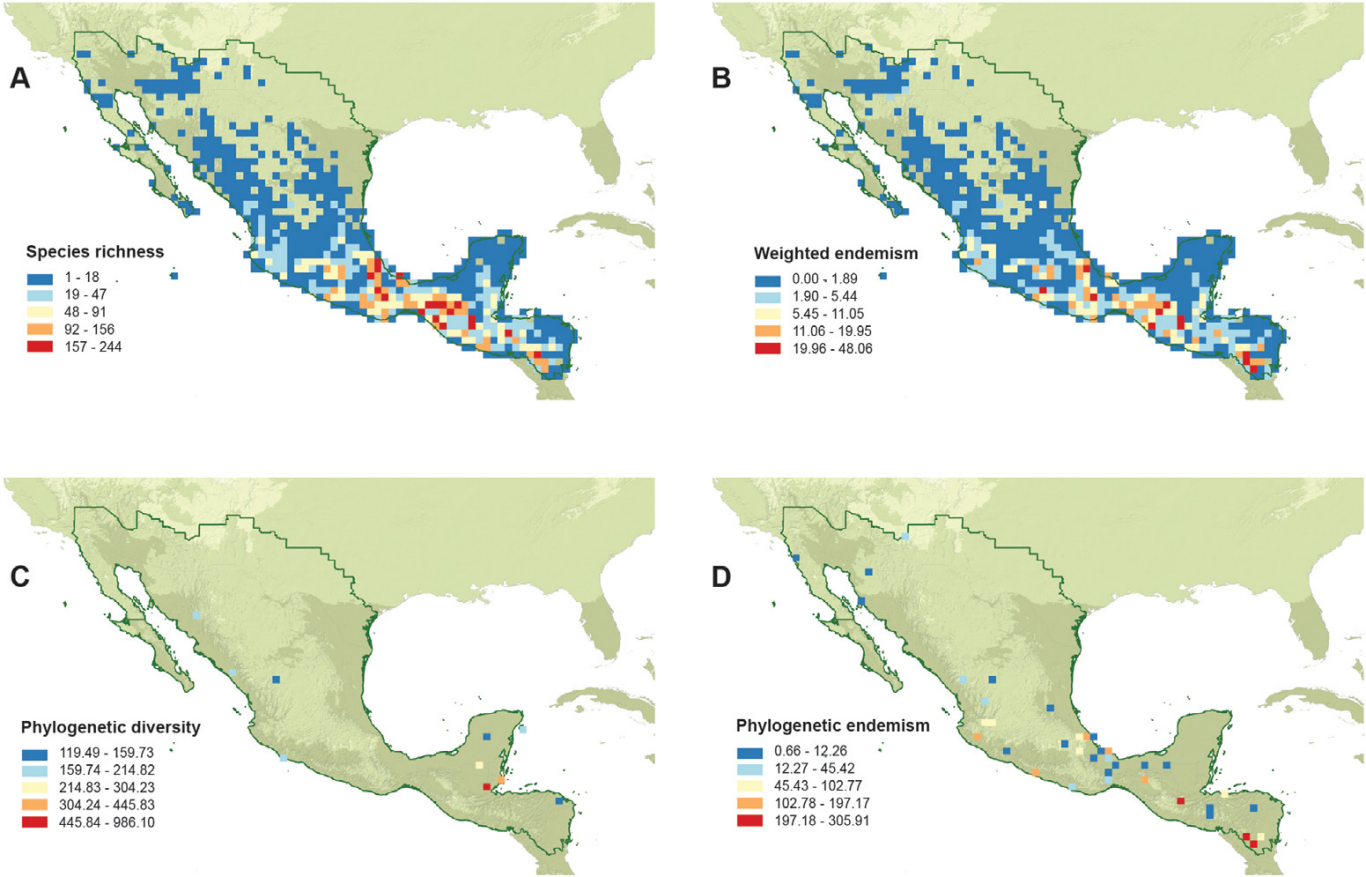
Evolutionary biodiversity patterns of orchids distributed in Megamexico. **A.** Species richness. **B.** Weighted endemism. **C.** Phylogenetic diversity. **D.** Phylogenetic endemism.

Estimates of species richness, weighted endemism, phylogenetic diversity, and phylogenetic endemism based on all orchid species without taking life form into account are shown in Fig. 2. Orchid species richness was highest in the Chiapas Highlands (Fig. 2A). The highest levels of weighted endemism were detected in eleven cells located in central Nicaragua, southeastern Mexico, and central Guatemala (Fig. 2B; Appendix E). Phylogenetic diversity was significantly higher in southeastern Mexico and Chiapas (Fig. 2C). In addition, phylogenetic diversity was elevated in several cells located in Veracruz, along the Gulf of Mexico, and in northern Oaxaca. High levels of phylogenetic endemism and weighted endemism were found in congruent areas (Fig. 2D); however, the areas with high levels of weighted endemism were smaller. The localities, number of species, as well as the genera and corresponding subtribes for areas with high levels of weighted and phylogenetic endemism are listed in Appendix E.

We found that both the number of cells and the levels of weighted endemism, phylogenetic endemism, and phylogenetic diversity differed throughout Megamexico. A frequency graph displaying ranges of non-endemic plants in Megamexico shows that the majority of non-endemic plants do not have extensive distributions outside this biogeographic province, with possibly no effect on estimations of weighted endemism, phylogenetic endemism, and phylogenetic diversity (Appendix F). The predominant species in the areas with the highest estimations of these measures belong to the subtribes Pleurothallidinae, Laeliinae, Oncidiinae and Maxillariinae.

### 3.4 Life forms and endemism

**Fig. 3:**
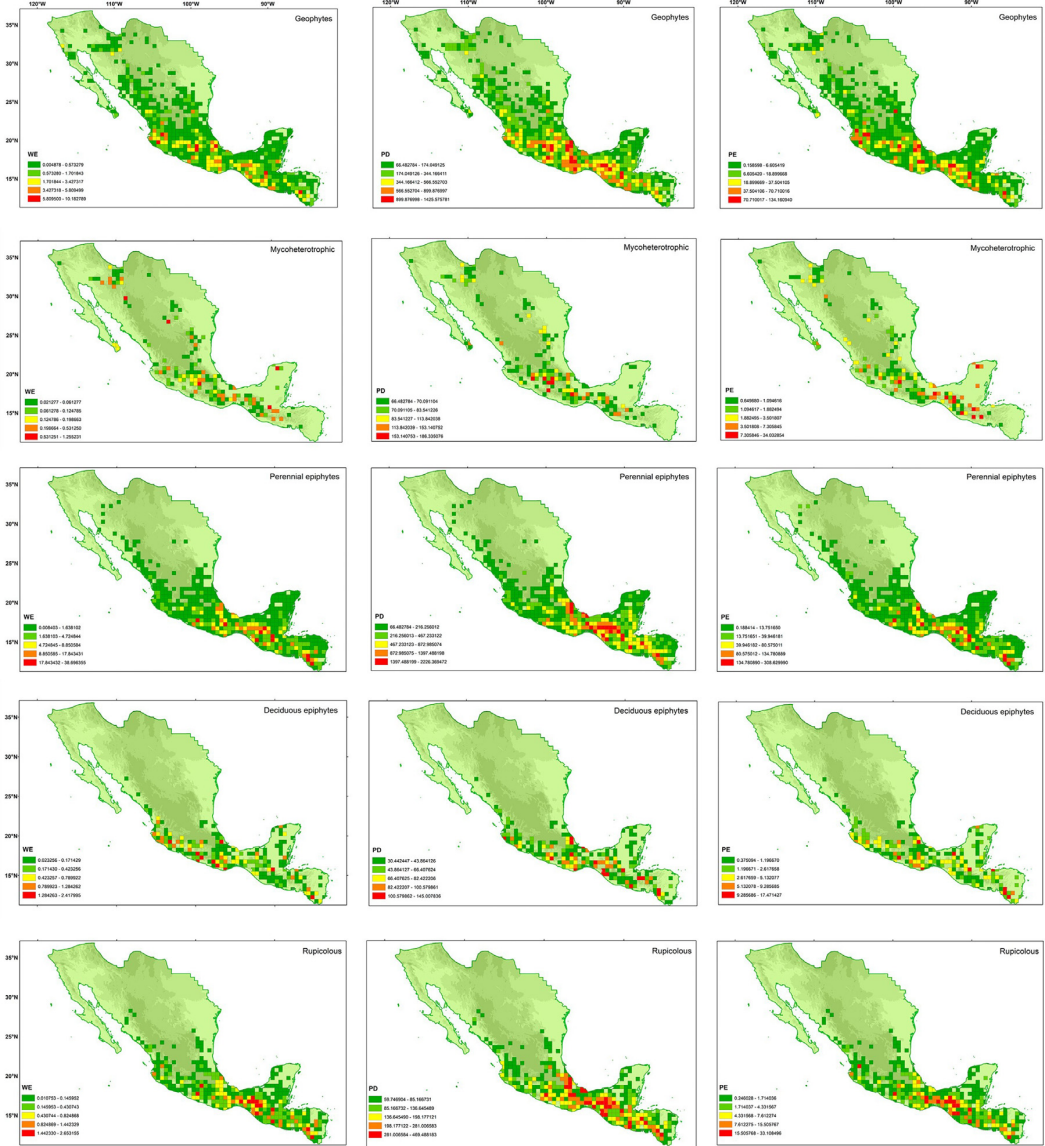
Weighted endemism, phylogenetic diversity, and phylogenetic endemism for every life form of the orchids in Megamexico (geophytes/perennial epiphytes/deciduous epiphytes/rupicolous/mycoheterotrophic).

**Fig. 4:**
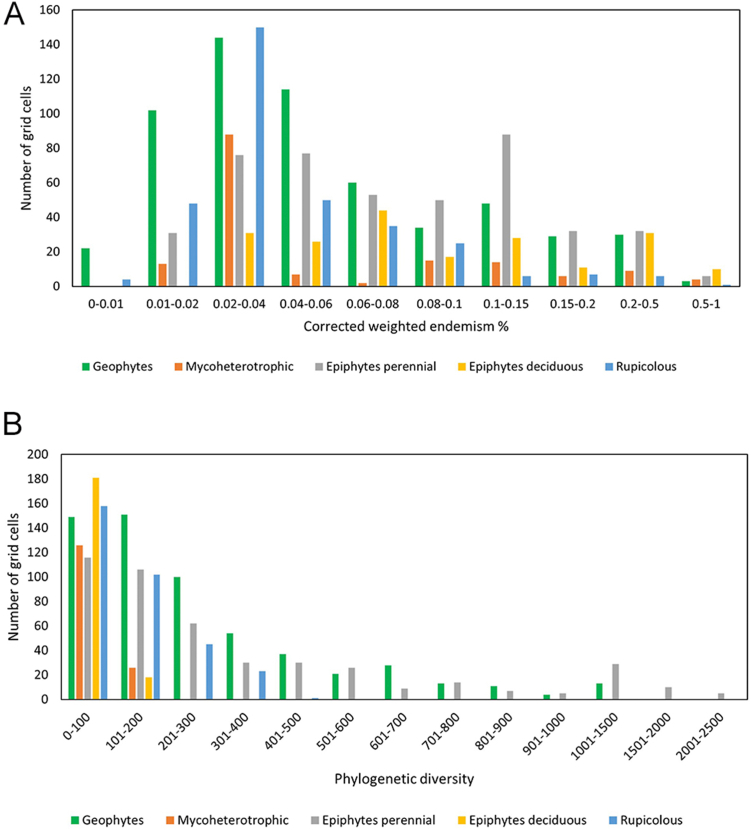
Frequency distribution histogram of **A.** corrected weighted endemism and **B.** phylogenetic diversity for every life form of the orchids of Megamexico (geophytes/mycoheterotrophic/perennial epiphytes/deciduous epiphytes/rupicolous).

Estimates of weighted endemism, phylogenetic diversity, and phylogenetic endemism according to life forms are shown in Fig. 3. The extent of areas of endemism for the orchids of Megamexico varied according to life form. Mycoheterotrophic life forms were associated with the smallest areas of endemism, whereas perennial epiphytes occupied the largest areas. Deciduous epiphytes in some areas in the Yucatan Peninsula had high levels of weighted and phylogenetic endemism, whereas other life forms in the same region did not. Perennial epiphytes were present in areas of elevated weighted endemism and phylogenetic endemism across the topographically complex mountain chains of northern Central America and southern Mexico. Geophytes and mycoheterotrophs were detected in a few areas of weighted and phylogenetic endemism in deserts such as the Chihuahuan Desert. For all life forms, the largest areas that showed weighted or phylogenetic endemism occurred in topographically complex regions, with the majority located in southern Mexico and northern Central America. Fig. 4 shows the corrected weighted endemism corresponding to life form.

## 4 Discussion

### 4.1 Phylogenetic analyses

The evaluation of the spatial patterns of phylogenetic diversity and phylogenetic endemism provide an innovative continuous estimate of land protection status, fine-scale data in untouched regions that can be utilized to detect complementary priority areas that contain concentrations of taxa that are evolutionarily singular, vulnerable due to their small range size and/or poorly protected across their distributions (Kling et al., 2018). Therefore, based on our findings on the endemism, phylogenetic diversity, and phylogenetic endemism for the orchids of Megamexico we suggest that the areas located at the southern edge of this biogeographic province—many of which are being transformed for agricultural use—should be proposed as complementary areas for conservation.

There has been much debate about the accuracy of approaches for reconstructing large-scale evolutionary trees in spatial phylogenetic studies utilizing systems software to reconstruct a phylogeny. Among the arguments against are the inadequate representativeness of the taxa of interest in gene databases for projects in which no sequencing was carried out. However, empirical studies have shown that adequate evolutionary trees can be retrieved in spite of sampling incompleteness, molecular markers, and methods, and that estimates of phylogenetic diversity show insignificant differences (Soltis and Soltis, 2016; Allen et al., 2019; Mishler et al., 2020). In our phylogeny of the orchids of Megamexico, the lack of sequences did not have any effect on the topology, as we followed the strategy of adding these species as polytomies at the midpoint of the genus branch length (Jin and Qian, 2019). Indeed, the phylogeny retrieved here coincided with previous estimates of phylogenetic relationships in the Orchidaceae (Freudenstein and Chase, 2015; Givnish et al., 2015, 2016; Li et al., 2019).

### 4.2 Areas of endemism and phylogenetic endemism

The highest estimates of species richness, weighted endemism, phylogenetic diversity, and phylogenetic endemism were similarly distributed in mountainous terrain, mostly in areas associated with the Trans-Mexican Volcanic Belt, the Sierra Madre Occidental, the Sierra Madre Oriental, Chiapas Highlands, Sierra Madre del Sur, and the Sierra de Guatemala. Previous estimates either with the entire vascular flora of Mexico (Sosa et al., 2018), or of specific taxonomic or functional groups identified similar areas of endemism (ferns: Sanginés–Franco et al., 2015; gymnosperms: Contreras–Medina and Luna–Vega, 2007; oaks: Rodríguez–Correa et al., 2015; grasses: Dávila–Aranda et al., 2004; bromeliads and orchids: Estrada–Sánchez et al., 2019; geophytes: Cuéllar–Martínez and Sosa, 2016).

In southeastern Mexico and northern Central America, estimates of phylogenetic diversity were higher and occupied more grids than those of weighted endemism. Our study also found that phylogenetic diversity tends to be high in topographically complex areas, which is consistent with findings from studies around the world (Mastretta–Yanes et al., 2015; Mráz et al., 2016; Baldwin, 2019). Thus, our work corroborates the hypothesis that phylogenetic diversity and endemism are elevated in mountain systems. Remarkably, many of the areas we identified as having high levels of endemism and phylogenetic endemism have been previously proposed as refugia. For example, we found high levels of endemism in Córdoba, Sierra de Juárez, and Soconusco, areas that Toledo (1982) proposed as refugia based on tree diversity and endemic species of Mexico and Guatemala. Furthermore, our study indicated that endemism was high in the tropical rainforests of Oaxaca and Chiapas, areas that have been previously recognized as refugia on karst soils (Wendt, 1989). These areas are important not only because of their elevated diversity but also for their high levels of endemism.

### 4.3 Life forms and lineages

**Fig. 5:**
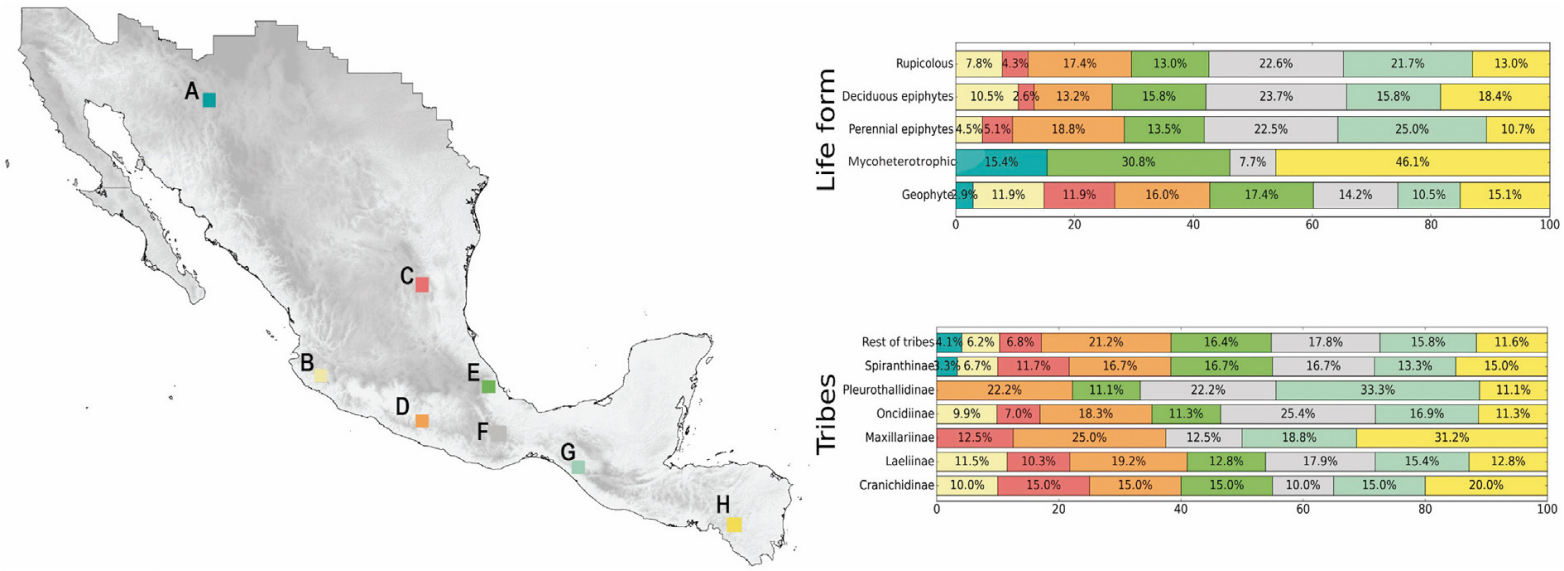
Map showing only the areas of Megamexico where the estimates of weighted endemism, phylogenetic diversity, and phylogenetic endemism are highest (grids A-H). The frequency of life forms and tribes is on the right, expressed as a percentage. Bar colors corresponding to the grids in the map (A-H).

When life form was included in our analyses, interesting patterns of weighted endemism, phylogenetic diversity, and phylogenetic endemism were identified (Fig. 4, Fig. 5). In the majority of areas in which endemism and phylogenetic endemism were estimated to be high, epiphytes were the predominant life form (Fig. 4, Fig. 5). This finding is not surprising as 69% of the total number of orchid species in the world are epiphytes (Zotz, 2013). Areas dominated by epiphytes are mainly located in mountain systems on the Pacific coastal regions of Mexico and Central America, such as the Sierra Madre Occidental, Sierra Madre del Sur and in smaller sierras in northern Central America. The epiphytes found in these areas include species of Laeliinae, Maxillarinae, Oncidiinae and Pleurothallidinae (Fig. 5). Epiphytic orchid species richness has been linked to bark texture and height of trees in the cloud forests of the Pacific slopes of Mexico, particularly in Oaxaca (Hernández–Pérez et al., 2018). This relationship between tree attributes and epiphytic species richness suggests that trees generate diverse microhabitats and that large trees provide extended time for the establishment of orchids. This conclusion is supported by research on the cloud forests of eastern Brazil, where orchid epiphyte establishment depends more on bark attributes than on phorophyte species (Francisco et al., 2021). We also found that the majority of orchid geophytes — mostly Cranichidinae and Spiranthinae species — are located in areas of endemism with drier conditions (Fig. 4, Fig. 5).

Our analysis also revealed that areas in Megamexico with high orchid endemism and phylogenetic endemism host species from distant lineages, corroborating the hypothesis that filtering processes select for species that are more distantly related and characterized by certain highly preserved functional traits adapted to the stable climatic conditions inside areas of endemism or refugia (Molina–Venegas et al., 2015). Epiphytic species in areas of high phylogenetic diversity and endemism comprised different lineages; for example, Pleurothallidinae and Laeliinae are closely related, whereas Oncidiinae and Maxillarinae are distantly related lineages (Givnish et al., 2016).

### 4.4 Conservation

**Fig. 6:**
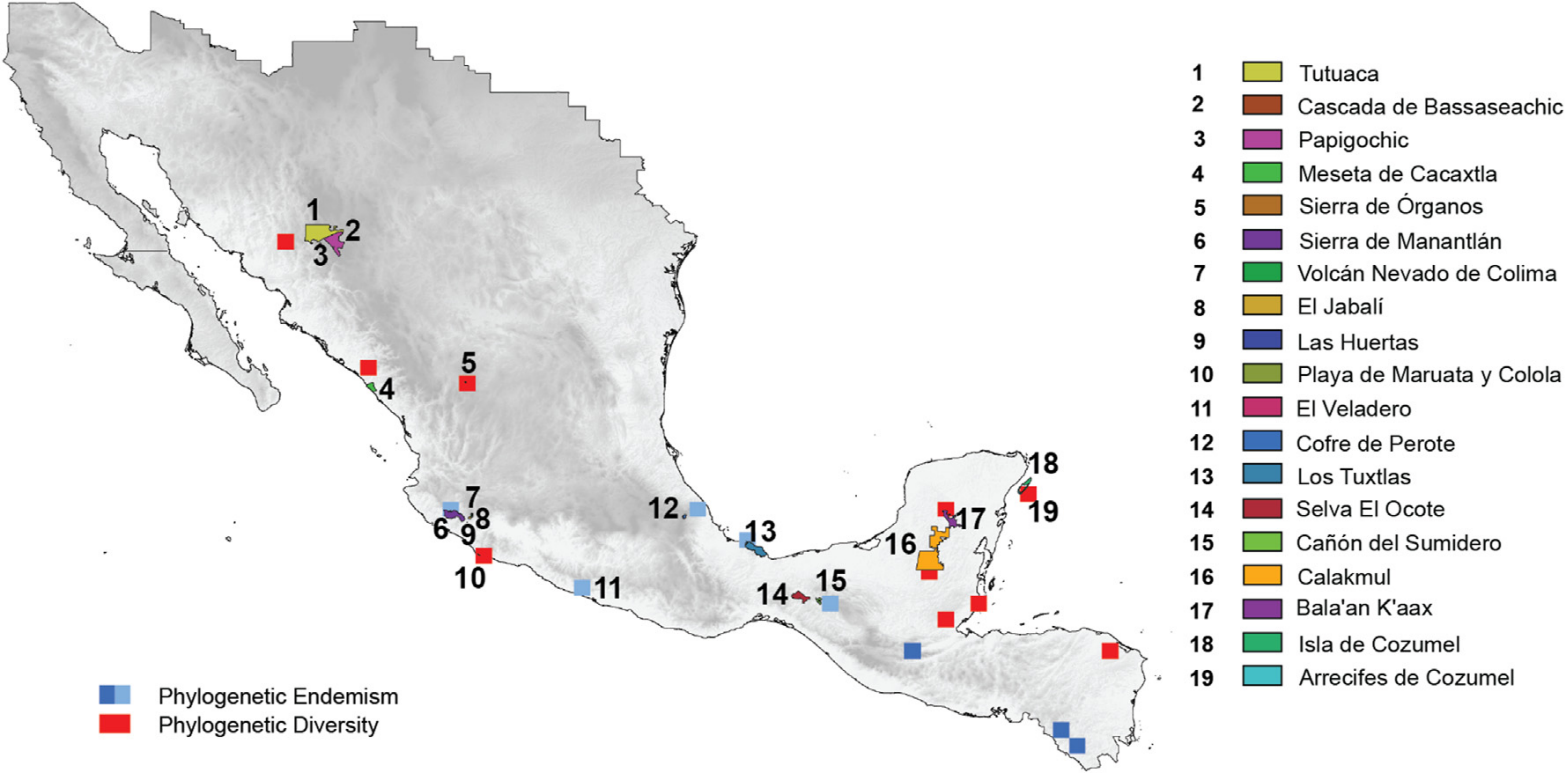
Map of the system of natural protected areas of Mexico combined with areas of elevated phylogenetic endemism and phylogenetic diversity to show that many of the areas with high phylogenetic diversity and phylogenetic endemism are not located in protected areas. The names of the areas follow the National Commission of Protected Areas (https://www.gob.mx/conanp#1692).

The Natural Protected Areas System of Mexico (http://sig.conanp.gob.mx/website/pagsig/listanp/) designates 190 protected areas, of which twenty are important because of their considerable area and the type of ecosystems they preserve (Fig. 6). However, zones with high phylogenetic diversity and phylogenetic endemism for orchids of Megamexico are mostly located outside protected areas (Fig. 6). There are some exceptions, however, such as the Sierra de Manantlán, which is situated on the Pacific slopes of Mexico and coincides with significant phylogenetic endemism (Fig. 6). In addition, a few reserves on the Yucatan Peninsula, such as Calakmul and Bala'an Kaax, are in important areas of phylogenetic diversity. Protected areas with tropical rainforests such as Selva del Ocote and Los Tuxtlas also coincide with areas of phylogenetic endemism (Fig. 6). Nevertheless, most areas of high endemism and phylogenetic endemism fall in unprotected regions. Moreover, three of the most important areas of phylogenetic endemism for orchids, located in the southern extreme of Megamexico, are notably unprotected: the cloud forests and tropical forests of Chiapas and northern Nicaragua. These important areas of orchid endemism must be protected.

Currently, more than ten orchid species are extinct in Mexico, and with the exception of one terrestrial orchid, all are epiphytes (Soto–Arenas et al., 2007). Previous studies have already demonstrated that clearing and fragmentation of montane forests have negative impacts on orchids in Chiapas (Cayuela et al., 2006). Thus, habitat loss has to be evaluated to select the most threatened areas containing the greatest phylogenetic diversity and endemism. Furthermore, orchid conservation strategies must consider not only protecting these areas but also multiple factors that influence the distribution and permanence of species. Orchids rely on extrinsic factors, such as interactions with other organisms, for their survival; they are sensitive to environmental changes either directly or indirectly through the organisms with which they interact, such as mycorrhizal fungi (Phillips et al., 2011; Reiter et al., 2018), or pollinators (Micheneau et al., 2009).

## 5 Conclusions

Areas of endemism and phylogenetic endemism for the orchids of Megamexico were identified in the rugged topography of mountain ranges, the majority in southernmost Megamexico. These areas contain diverse microclimates with many habitats especially suitable for epiphytes, including tribes such as Oncidiinae, Maxillarinae and Pleurothallidinae. Our results also corroborate the hypothesis that distantly related species share space in areas of endemism and phylogenetic endemism. We recommend that these areas of high endemism and phylogenetic endemism be designated priority areas for conservation.

## Author contributions

V.S. and B.E.G.R. designed the study; B.E.G.R. and M.V.C. performed analyses; B.E.G.R. and V.S. collected data; V.S. and B.E.G.R. led the writing of the manuscript. All authors contributed critically to the drafts and gave final approval for publication.

## Declaration of competing interest

The authors declare that they have no conflicts of interest.

## Supplementary data

**Appendix A.** Bibliographic sources for the life forms of the orchids of Megamexico. **Appendix B.** Database of the Orchidaceae of Megamexico including life form. **Appendix C.** Phylogenetic estimation of the 1732 orchids present in Megamexico using the method of Jin and Qian (2019). The numbers in the branches represent the age of the nodes. The grouping by color is done at the subtribe level. The subtribes in yellow belong to the Vanilloideae subfamily, in red to Cypripedioideae, in blues to Orchidoideae and in greens and pink to Epidendroideae. **Appendix D.** Corrected weighted endemism (CWE) of orchids of Megamexico. **Appendix E.** Grid cells with a high rate of endemism of the Orchidaceae of Megamexico. **Appendix F.** Frequency histogram of the ranges of species in km^2^ with distribution beyond Megamexico. Multimedia component 1
