# Supplementary material for: Phylogenetic endemism of the orchids of Megamexico reveals complementary areas for conservation
Source: Plant Divers. 2022 Mar 25;44(4):351–9. doi: 10.1016/j.pld.2022.03.004 (PMC9363653; doi:10.1016/j.pld.2022.03.004)
Supplement: Supplementary file 1 — Appendix A. Bibliographic sources for the life forms of the orchids of Megamexico. Appendix B. Database of the Orchidaceae of Megamexico including life form. Appendix C. Phylogenetic estimation of the 1732 orchids present in Megamexico using the method of Jin and Qian (2019). The numbers in the branches represent the age of the nodes. The grouping by color is done at the subtribe level. The subtribes in yellow belong to the Vanilloideae subfamily, in red to Cypripedioideae, in blues to Orchidoideae and in greens and pink to Epidendroideae. Appendix D. Corrected weighted endemism (CWE) of orchids of Megamexico. Appendix E. Grid cells with a high rate of endemism of the Orchidaceae of Megamexico. Appendix F. Frequency histogram of the ranges of species in km2 with distribution beyond Megamexico. [file mmc1.zip › Appendix A. Specialized bibliography.docx]

**Appendix A.** References used to obtain information on the life forms of the orchids of Megamexico.

Ames, O. & D. Stewart-Correll. 1985. Orchids of Guatemala and Belize. Dover Publications Inc. New York. 780 pp.

Benzing, D. H. 1987. Vascular epiphytism: taxonomic participation and adaptive diversity. Annals of the Missouri Botanical Garden. 74: 183–204. doi:10.2307/2399394

Benzing, D. H. 1990. Epiphytism: a preliminary overview. In Vascular epiphytes. General biology and related biota (ed. P. S. Ashton, S. P. Hubbell, D. H. Janzen, A. G. Marshall, P. H. Raven & P. B. Tomlinson), pp. 1–42. Cambridge University Press.

Beutelspacher-Braigts, C. R. & I. Moreno-Molina. 2018. Las Orquídeas de Chiapas. Instituto Chinoin. México. 640 pp.

Dressler, R. L. 1981. The Orchids: Natural History and Classification. Harvar University Press, Cambridge. 332 pp.

Dressler, R. L. & G. E. Pollard. 1974. El género *Encyclia* en México. Asociación Mexicana de Orquideología, México. 158 pp.

González-Tamayo, J. R. & L. Hernández-Hernández. 2010. Las orquídeas del Occidente de México. COECYTJAL. México. 304 pp.

Gravendeel, B., Smithson, A., Slik, F. J. W., & Schuiteman, A. 2004. Epiphytism and pollinator specialization: Drivers for orchid diversity? Philosophical Transactions of the Royal Society B: Biological Sciences, 359: 1523–1535. doi:10.1098/rstb.2004.1529

Hágsater, E., M. Á. Soto-Arenas, G. A. Salazar-Chávez, R. Jiménez-Machorro, M. A. López-Rosas y R. L. Dressler. 2005. Las orquídeas de México. Instituto Chinonin, México, 304 pp.

Halbinger, F., & Soto, M. 1997. Laelias of Mexico. Orquídea (México, DF), 15: 1-160.

Hágsater, E. y G. A. Salazar. 1990. Icones orchidacearum Fascicle 1. Part 1. Orchids of Mexico. Asociación Mexicana de Orquideología. México.

Hágsater, E. 1993. Icones orchidacearum. Fascicle 2. The genus *Epidendrum* Part 1. A Century of New Species in Epidendrum. Asociación Mexicana de Orquideología. México.

Hágsater, E. 1999. Icones orchidacearum. Fascicle 3. The genus *Epidendrum* Part 2. A Second Century of New Species in *Epidendrum*. Asociación Mexicana de Orquideología. México.

Hágsater, E. 2001. Icones orchidacearum. Fascicle 4. The genus *Epidendrum* Part 3. A third Century of New Species in Epidendrum. Asociación Mexicana de Orquideología. México.

Hágsater, E. y M. A. Soto. 2003. Icones orchidacearum Fascicle 5 y 6. Part 2 y 3. Orchids of Mexico. Asociación Mexicana de Orquideología. México.

Hágsater, E. 2004. Icones orchidacearum. Fascicle 7. The genus *Epidendrum* Part 4. A fourth Century of New Species in *Epidendrum*. Asociación Mexicana de Orquideología. México.

Hágsater, E. 2006. Icones orchidacearum. Fascicle 8. The genus *Epidendrum* Part 5. Species New and old in Epidendrum. Asociación Mexicana de Orquideología. México.

Hágsater, E. 2007. Icones orchidacearum. Fascicle 9. The genus *Epidendrum* Part 6. Species New and old in Epidendrum. Asociación Mexicana de Orquideología. México.

Hágsater, E. y M. A. Soto. 2008. Icones orchidacearum. Fascicle 10. Part 4. Orchids of Mexico. Asociación Mexicana de Orquideología. México.

Hágsater, E. 2008. Icones orchidacearum. Fascicle 11. The genus *Epidendrum* Part 7. Species New and old in *Epidendrum*. Asociación Mexicana de Orquideología. México.

Hágsater, E. 2009. Icones orchidacearum. Fascicle 12. The genus *Epidendrum* Part 8. Species New and old in *Epidendrum*. Asociación Mexicana de Orquideología. México.

Hágsater, E. 2010. Icones orchidacearum. Fascicle 13. The genus *Epidendrum* Part 9. Species New and old in *Epidendrum*. Asociación Mexicana de Orquideología. México.

Hágsater, E. 2013. Icones orchidacearum. Fascicle 14. The genus *Epidendrum* Part 10. Species New and old in *Epidendrum*. Asociación Mexicana de Orquideología. México.

Hágsater, E. 2015. Icones orchidacearum. Fascicle 15(1). The genus *Epidendrum* Part 11. Species New and old in *Epidendrum*. Asociación Mexicana de Orquideología. México.

Hágsater, E. 2016. Icones orchidacearum. Fascicle 15(2). The genus *Epidendrum* Part 11. Species New and old in *Epidendrum*. Asociación Mexicana de Orquideología. México.

Hágsater, E. & E. Santiago. 2018. Icones orchidacearum. Fascicle 16(1). The genus *Epidendrum* Part 12. Species New and old in *Epidendrum*. Asociación Mexicana de Orquideología. México.

Hágsater, E. & E. Santiago. 2018. Icones orchidacearum. Fascicle 16(2). The genus *Epidendrum* Part 12. Species New and old in *Epidendrum*. Asociación Mexicana de Orquideología. México.

Hágsater, E. & E. Santiago. 2019. Icones orchidacearum. Fascicle 17(1). The genus *Epidendrum* Part 13. Species New and old in *Epidendrum*. Asociación Mexicana de Orquideología. México.

Hágsater, E. & E. Santiago. 2020. Icones orchidacearum. Fascicle 17(2). The genus *Epidendrum* Part 13. Species New and old in *Epidendrum*. Asociación Mexicana de Orquideología. México.

Hágsater, E. & E. Santiago. 2020. Icones orchidacearum. Fascicle 18(1). The genus *Epidendrum* Part 14. Species New and old in *Epidendrum*. Asociación Mexicana de Orquideología. México.
