# Supplementary material for: Phylogenetic endemism of the orchids of Megamexico reveals complementary areas for conservation
Source: Plant Divers. 2022 Mar 25;44(4):351–9. doi: 10.1016/j.pld.2022.03.004 (PMC9363653; doi:10.1016/j.pld.2022.03.004)
Supplement: Supplementary file 1 — Appendix A. Bibliographic sources for the life forms of the orchids of Megamexico. Appendix B. Database of the Orchidaceae of Megamexico including life form. Appendix C. Phylogenetic estimation of the 1732 orchids present in Megamexico using the method of Jin and Qian (2019). The numbers in the branches represent the age of the nodes. The grouping by color is done at the subtribe level. The subtribes in yellow belong to the Vanilloideae subfamily, in red to Cypripedioideae, in blues to Orchidoideae and in greens and pink to Epidendroideae. Appendix D. Corrected weighted endemism (CWE) of orchids of Megamexico. Appendix E. Grid cells with a high rate of endemism of the Orchidaceae of Megamexico. Appendix F. Frequency histogram of the ranges of species in km2 with distribution beyond Megamexico. [file mmc1.zip › Appendix E..docx]

**Appendix E.** Grid cells with a high rate of endemism of the Orchidaceae of Megamexico. The number in parentheses indicates the species of each subtribe that are present in the cell.

| **Grid**  **Cell** | **Location** | **Number of records** | **Number of species** | **Genera present** | **Orchid lineages** |
| --- | --- | --- | --- | --- | --- |
| 1 | Nicaragua (Jonotega-Matagalpa) | 479 | 239 | *Acianthera*, *Anathallis*, *Arpophyllum*, *Aspidogyne*, *Aulosepalum*, *Barbosella, Barkeria*, *Brassavola, Brassia, Bulbophyllum, Campylocentrum*, *Caularthron*, *Chysis*, *Coccineorchis*, *Cochleanthes*, *Comparettia*, *Cranichis, Cyclopogon, Cycnoches, Cyrtochiloides, Dichaea, Dimerandra, Dracula, Dressleria, Elleanthus*, *Encyclia*, *Epidendrum*, *Erycina, Galeandra, Gongora, Goodyera, Govenia, Greenwoodiella, Habenaria, Helleriella, Isochilus, Jacquinella, Lacaena, Laelia, Leochilus, Lepanthes, Liparis, Lockhartia, Lycaste, Malaxis, Masdevallia, Maxillaria*, *Muscarella*, *Myoxanthus, Nemaconia, Nidema, Octomeria, Oncidium, Ornithocephalus, Pelexia, Platystele, Pleurothallis*, *Polystachya, Ponthieva, Prescottia, Prosthechea*, *Restrepia, Restrepiella, Rhynchostele, Rossioglossum, Sacoila, Sarcoglottis, Scaphosepalum, Scaphyglottis, Sobralia, Specklinia, Stanhopea, Stelis, Stenorrhynchos, Stenotyla, Trichocentrum, Trichopilia, Trichosalpinx, Xylobium* | Laeliinae (63) Pleurothallidinae (52) Maxillariinae (28) Oncidiinae (25) Spiranthinae (11) Stanhopeinae (9) Sobraliinae (8) Zygopetalinae (8) Ponerinae (7)  Dendrobiinae (4) Goodyerinae (4) Malaxidinae (4) Orchidiinae (4) Sobralieae (3) Angraecinae (2) Calypsoinae (2) Catasetinae (2) Bletiinae (1) Cranichidinae (1) Polystachyinae (1) |
| 2 | Guatemala (Alta Verapaz-Baja Verapaz) | 521 | 181 | *Acianthera, Anathallis, Arpophyllum, Aspidogyne, Aulosepalum, Bletia, Brassavola, Brassia, Calanthe, Campylocentrum, Coelia, Comparettia, Corymborkis, Cuitlauzina, Cyclopogon, Dichaea, Dichromanthus, Dinema, Domingoa, Dracula, Elleanthus, Encyclia, Epidendrum, Govenia, Habenaria, Hapalorchis, Homalopetalum, Isochilus, Jacquinella, Lacaena, Leochilus, Lepanthes, Liparis, Lockhartia, Lycaste, Malaxis, Masdevallia, Maxillaria, Microchilus, Muscarella, Oncidium, Ornithocephalus, Phragmipedium, Platystele, Pleurothallis, Ponthieva, Prescottia, Prosthechea, Psilochilus, Rhynchostele, Sacoila, Scaphyglottis, Sobralia, Specklinia, Stelis, Stenorrhynchos, Stenotyla, Telipogon, Trichosalpinx, Xylobium* | Pleurothallidinae (56) Laeliinae (41) Oncidiinae (19) Maxillariinae (10) Spiranthinae (10) Orchidiinae (9) Zygopetalinae (7) Malaxidinae (5) Cranichidinae (5) Calypsoinae (3) Sobraliinae (3) Ponerinae (2) Sobralieae (2) Goodyerinae (2) Angraecinae (1) Bletiinae (1) Collabiinae (1) Phragmipediinae (1) Stanhopeinae (1) Triphorinae (1) Tropidiinae (1) |
| 3 | Mexico (Chiapas, on the border with Guatemala) | 837 | 241 | *Acianthera, Anathallis, Arpophyllum, Aulosepalum, Barkeria, Bletia, Brassavola, Brassia, Campylocentrum, Catasetum, Caularthron, Clowesia, Coelia, Comparettia, Corallorhiza, Cranichis, Cryptarrhena, Cuitlauzina, Cyclopogon, Cycnoches, Cyrtochiloides, Cyrtopodium, Deiregyne, Dichaea, Dichromanthus, Dinema, Domingoa, Elleanthus, Encyclia, Epidendrum, Erycina, Eulophia, Fernandezia, Funkiella, Galeottiella, Gongora, Goodyera, Govenia, Guarianthe, Habenaria, Isochilus, Jacquinella, Kefersteinia, Kionophyton, Leochilus, Lepanthes, Liparis, Lockhartia, Lycaste, Macroclinium, Malaxis, Masdevallia, Maxillaria, Meiracyllium, Mesadenella, Microthelys, Mormodes, Muscarella, Nemaconia, Nidema, Notylia, Oncidium, Ornithocephalus, Pelexia, Platystele, Plectrophora, Pleurothallis, Polystachya, Ponthieva, Prosthechea, Restrepia, Restrepiella, Rhynchostele, Rossioglossum, Scaphyglottis, Sobralia, Specklinia, Stanhopea, Stelis, Stenorrhynchos, Tamayorkis, Trichocentrum, Trichopilia, Trichosalpinx, Vanilla* | Laeliinae (55) Pleurothallidinae (47) Oncidiinae (39) Maxillariinae (19) Spiranthinae (14) Orchidiinae (11) Zygopetalinae (8) Malaxidinae (7) Cranichidinae (6) Calypsoinae (6) Ponerinae (5) Bletiinae (4) Catasetinae (4) Angraecinae (2) Polystachyinae (2) Sobralieae (2) Sobraliinae (2) Stanhopeinae (2) Vanillinae (2) Cyrtopodiinae (1) Eulophiinae (1) Galeottiellinae (1) Goodyerinae (1) |
| 4 | Guatemala (Alta Verapaz) | 333 | 198 | *Acianthera, Anathallis, Aspidogyne, Aulosepalum, Barkeria, Beloglottis, Bletia, Brachystele, Brassia, Bulbophyllum, Calanthe, Campylocentrum, Caularthron, Chysis, Coelia, Coenoemersa, Comparettia, Corallorhiza, Corymborkis, Cranichis, Cuitlauzina, Cyclopogon, Deiregyne, Dichaea, Dichromanthus, Dinema, Domingoa, Dryadella, Encyclia, Epidendrum, Erycina, Funkiella, Gongora, Goodyera, Govenia, Guarianthe, Habenaria, Hexalectris, Isochilus, Jacquinella, Kreodanthus, Lacaena, Lepanthes, Liparis, Lockhartia, Lycaste, Malaxis, Masdevallia, Maxillaria, Mesadenella, Microchilus, Microthelys, Mormodes, Muscarella, Nidema, Oncidium, Pelexia, Platystele, Pleurothallis, Polystachya, Ponthieva, Prescottia, Prosthechea, Psilochilus, Restrepia, Restrepiella, Rhynchostele, Rossioglossum, Sarcoglottis, Scaphyglottis, Sobralia, Specklinia, Stanhopea, Stelis, Trichocentrum, Trichosalpinx, Tropidia, Vanilla* | Pleurothallidinae (47) Laeliinae (46) Spiranthinae (15) Orchidiinae (14) Maxillariinae (13) Oncidiinae (11) Goodyerinae (7) Cranichidinae (6) Malaxidinae (6)  Bletiinae (5) Calypsoinae (4) Zygopetalinae (4) Angraecinae (3) Sobraliinae (3) Stanhopeinae (3) Dendrobiinae (2) Ponerinae (2) Tropidiinae (2) Catasetinae (1) Collabiinae (1) Polystachyinae (1) Triphorinae (1) Vanillinae (1) |
| 5 | Nicaragua (Matagalpa-Boaco) | 228 | 136 | *Acianthera, Arpophyllum, Aulosepalum, Barkeria, Beloglottis, Brassia, Calanthe, Corymborkis, Cranichis, Cycnoches, Dichaea, Dimerandra, Dinema, Dracula, Dryadella, Elleanthus, Encyclia, Epidendrum, Erycina, Gongora, Govenia, Guarianthe, Habenaria, Helleriella, Houlletia, Jacquinella, Leochilus, Lepanthes, Lepanthopsis, Lockhartia, Lycaste, Malaxis, Masdevallia, Maxillaria, Muscarella, Myoxanthus, Myrmecophila, Nemaconia, Nidema, Oestlundia, Oncidium, Ornithocephalus, Pelexia, Platystele, Pleurothallis, Pleurothallopsis, Polystachya, Ponthieva, Prosthechea, Scaphyglottis, Sobralia, Specklinia, Stanhopea, Stelis, Telipogon, Trichocentrum, Trichopilia, Trichosalpinx, Xylobium, Zootrophion* | Laeliinae (39) Pleurothallidinae (34) Maxillariinae (15) Oncidiinae (10) Orchidiinae (7) Spiranthinae (5) Sobralieae (4) Stanhopeinae (4) Cranichidinae (3) Sobraliinae (3) Zygopetalinae (3) Polystachyinae (2) Ponerinae (2) Calypsoinae (1) Catasetinae (1) Collabiinae (1) Malaxidinae (1) Tropidiinae (1) |
| 6 | Mexico (Chiapas) | 1103 | 245 | *Anathallis, Arpophyllum, Aspidogyne, Aulosepalum, Barkeria, Bletia, Brachystele, Brassia, Calanthe, Campylocentrum, Chysis, Coelia, Corallorhiza, Corymborkis, Cranichis, Cuitlauzina, Cyclopogon, Dichaea, Dichromanthus, Dinema, Domingoa, Dracula, Elleanthus, Encyclia, Epidendrum, Eriopsis, Gongora, Goodyera, Govenia, Guarianthe, Habenaria, Hapalorchis, Homalopetalum, Ionopsis, Isochilus, Jacquinella, Laelia, Leochilus, Lepanthes, Lockhartia, Lycaste, Malaxis, Masdevallia, Maxillaria, Muscarella, Myoxanthus, Nemaconia, Nidema, Oncidium, Ornithocephalus, Pelexia, Platystele, Pleurothallis, Ponera, Ponthieva, Prescottia, Prosthechea, Psilochilus, Restrepia, Restrepiella, Rhyncholaelia, Rhynchostele, Rossioglossum, Sarcoglottis, Scaphosepalum, Scaphyglottis, Schiedeella, Sobralia, Specklinia, Stanhopea, Stelis, Stenorrhynchos, Stenotyla, Trichocentrum, Trichopilia, Trichosalpinx, Xylobium* | Laeliinae (66) Pleurothallidinae (53) Maxillariinae (24) Oncidiinae (22) Spiranthinae (12) Orchidiinae (9)  Zygopetalinae (9) Ponerinae (8) Calypsoinae (7) Bletiinae (6) Cranichidinae (6) Stanhopeinae (5) Sobralieae (4) Goodyerinae (3) Malaxidinae (3) Sobraliinae (3) Angraecinae (1) Collabiinae (1) Eriopsidinae (1) Triphorinae (1) Tropidiinae (1) |
| 7 | Nicaragua (Chontales-Boaco) | 177 | 113 | *Acianthera, Anathallis, Aspasia, Aspidogyne, Beloglottis, Brassavola, Brassia, Caularthron, Corymborkis, Cranichis, Cyclopogon, Dichaea, Dimerandra, Dresslerella, Dressleria, Echinosepala, Elleanthus, Encyclia, Epidendrum, Eulophia, Gongora, Habenaria, Ionopsis, Isochilus, Jacquinella, Leochilus, Liparis, Lockhartia, Macroclinium, Masdevallia, Maxillaria, Myoxanthus, Myrmecophila, Nidema, Octomeria, Oeceoclades, Pleurothallis, Polystachya, Prescottia, Prosthechea, Rossioglossum, Sacoila, Sarcoglottis, Scaphyglottis, Sobralia, Specklinia, Stanhopea, Stelis, Trichocentrum, Trichopilia, Vanilla* | Laeliinae (30) Pleurothallidinae (22) Maxillariinae (14)  Oncidiinae (12) Polystachyinae (5) Sobraliinae (4) Spiranthinae (4) Zygopetalinae (4) Sobralieae (3) Cranichidinae (2) Eulophiinae (2) Goodyerinae (2) Orchidiinae (2)  Stanhopeinae (2) Vanillinae (2) Malaxidinae (1) Ponerinae (1) Tropidiinae (1) |
| 8 | Mexico (Veracruz) | 1252 | 214 | *Acianthera, Acineta, Anathallis, Beloglottis, Bletia, Brassavola, Brassia, Calanthe, Campylocentrum, Catasetum, Chysis, Coelia, Comparettia, Corymborkis, Cranichis, Cryptarrhena, Cyclopogon, Cycnoches, Cypripedium, Cyrtochilum, Cyrtopodium, Deiregyne, Dendrophylax, Dichaea, Dichromanthus, Dinema, Domingoa, Elleanthus, Encyclia, Epidendrum, Gongora, Goodyera, Govenia, Guarianthe, Habenaria, Homalopetalum, Ionopsis, Isochilus, Jacquinella, Laelia, Leochilus, Lepanthes, Lycaste, Malaxis, Masdevallia, Maxillaria, Mesadenus, Microchilus, Mormodes, Mormolyca, Muscarella, Myrmecophila, Nemaconia, Nidema, Notylia, Oeceoclades, Oestlundia, Oncidium, Ornithocephalus, Paphiopedilum, Pelexia, Platystele, Pleurothallis, Polystachya, Ponthieva, Prescottia, Prosthechea, Psilochilus, Restrepiella, Rhyncholaelia, Rhynchostele, Sacoila, Sarcoglottis, Scaphyglottis, Sobralia, Specklinia, Spiranthes, Stanhopea, Stelis, Stenorrhynchos, Tolumnia, Trichocentrum, Trichopilia, Trichosalpinx, Trigonidium, Tropidia, Vanilla* | Laeliinae (62) Pleurothallidinae (29) Oncidiinae (27) Spiranthinae (19) Maxillariinae (12) Orchidiinae (10) Bletiinae (6) Ponerinae (5) Zygopetalinae (5) Cranichidinae (4) Stanhopeinae (4) Vanillinae (4) Angraecinae (3) Calypsoinae (3) Catasetinae (3) Goodyerinae (3) Cypripediinae (2) Cyrtopodiinae (2) Sobralieae (2) Tropidiinae (2)  Collabiinae (1) Eulophiinae (1) Malaxidinae (1) Paphiopedilinae (1) Polystachyinae (1) Sobraliinae (1) Triphorinae (1) |
| 9 | Mexico (Oaxaca) | 702 | 182 | *Acianthera, Anathallis, Arpophyllum, Aulosepalum, Barkeria, Bletia, Brassia, Bulbophyllum, Calanthe, Catasetum, Coenoemersa, Comparettia, Corallorhiza, Cyclopogon, Cypripedium, Deiregyne, Dichaea, Dichromanthus, Domingoa, Elleanthus, Encyclia, Epidendrum, Erycina, Funkiella, Galeoglossum, Gongora, Goodyera, Govenia, Habenaria, Homalopetalum, Isochilus, Jacquinella, Kreodanthus, Laelia, Lepanthes, Liparis, Malaxis, Maxillaria, Mesadenella, Microthelys, Mormodes, Nemaconia, Oncidium, Pleurothallis, Ponera, Ponthieva, Prescottia, Prosthechea, Rhynchostele, Sacoila, Sarcoglottis, Scaphyglottis, Schiedeella, Sobralia, Specklinia, Stanhopea, Stelis, Trichocentrum, Triphora* | Laeliinae (44) Pleurothallidinae (31) Spiranthinae (18) Oncidiinae (16) Malaxidinae (12) Calypsoinae (10) Ponerinae (8) Bletiinae (7) Maxillariinae (7) Orchidiinae (7) Cranichidinae (4) Zygopetalinae (4) Goodyerinae (3)  Catasetinae (2) Cypripediinae (2) Stanhopeinae (2) Collabiinae (1) Dendrobiinae (1) Sobralieae (1) Sobraliinae (1) Triphorinae (1) |
| 10 | Mexico (Guerrero) | 340 | 156 | *Acianthera, Anathallis, Artorima, Aulosepalum, Barkeria, Bletia, Bulbophyllum, Calanthe, Coenoemersa, Corallorhiza, Cranichis, Cyclopogon, Cypripedium, Cyrtopodium, Deiregyne, Dichaea, Dichromanthus, Domingoa, Encyclia, Epidendrum, Galeoglossum, Goodyera, Govenia, Habenaria, Helleriella, Hexalectris, Isochilus, Jacquinella, Kionophyton, Kraenzlinella, Laelia, Leochilus, Lepanthes, Liparis, Macroclinium, Malaxis, Maxillaria, Mesadenus, Microepidendrum, Mormodes, Nemaconia, Oestlundia, Oncidium, Ponera, Ponthieva, Prescottia, Prosthechea, Rhynchostele, Rossioglossum, Sacoila, Sarcoglottis, Sobralia, Stanhopea, Stelis, Stenorrhynchos, Trichocentrum, Trichosalpinx, Triphora* | Laeliinae (45) Oncidiinae (19) Pleurothallidinae (16) Spiranthinae (14) Bletiinae (13) Malaxidinae (9) Orchidiinae (7)  Calypsoinae (6) Cranichidinae (6) Maxillariinae (4) Ponerinae (4) Catasetinae (2)  Sobraliinae (2) Stanhopeinae (2) Collabiinae (1) Cypripediinae (1) Cyrtopodiinae (1) Dendrobiinae (1) Goodyerinae (1) Triphorinae (1) Zygopetalinae (1) |
| 11 | Mexico (Chiapas) | 2145 | 231 | *Acianthera, Anathallis, Arpophyllum, Aspidogyne, Bletia, Brassia, Bulbophyllum, Campylocentrum, Catasetum, Chysis, Coelia, Comparettia, Coryanthes, Cuitlauzina, Cyclopogon, Cycnoches, Dichaea, Dinema, Domingoa, Dryadella, Elleanthus, Encyclia, Epidendrum, Erycina, Eulophia, Gongora, Habenaria, Ionopsis, Isochilus, Jacquinella, Leochilus, Lepanthes, Liparis, Lockhartia, Lycaste, Malaxis, Masdevallia, Maxillaria, Mesadenella, Mormolyca, Muscarella, Myoxanthus, Nemaconia, Nidema, Notylia, Oeceoclades, Oncidium, Ornithocephalus, Pelexia, Platystele, Platythelys, Pleurothallis, Polystachya, Ponthieva, Prescottia, Prosthechea, Psilochilus, Restrepia, Restrepiella, Sacoila, Sarcoglottis, Scaphosepalum, Scaphyglottis, Schiedeella, Sobralia, Specklinia, Stanhopea, Stelis, Stenotyla, Trichocentrum, Trichopilia, Trichosalpinx, Trigonidium, Triphora, Tropidia, Vanilla* | Laeliinae (61) Pleurothallidinae (48) Maxillariinae (26) Oncidiinae (21) Spiranthinae (13) Stanhopeinae (9) Orchidiinae (5) Polystachyinae (5) Goodyerinae (4) Sobraliinae (4) Vanillinae (4) Bletiinae (3) Catasetinae (3) Dendrobiinae (3) Malaxidinae (3) Ponerinae (3) Sobralieae (3) Triphorinae (3) Cranichidinae (2) Eulophiinae (2) Angraecinae (2) Zygopetalinae (2) Calypsoinae (1) Gastrodiinae (1) |
