# Supplementary figures and images for: Phylogenetic endemism of the orchids of Megamexico reveals complementary areas for conservation
Source: Plant Divers. 2022 Mar 25;44(4):351–9. doi: 10.1016/j.pld.2022.03.004 (PMC9363653; doi:10.1016/j.pld.2022.03.004)

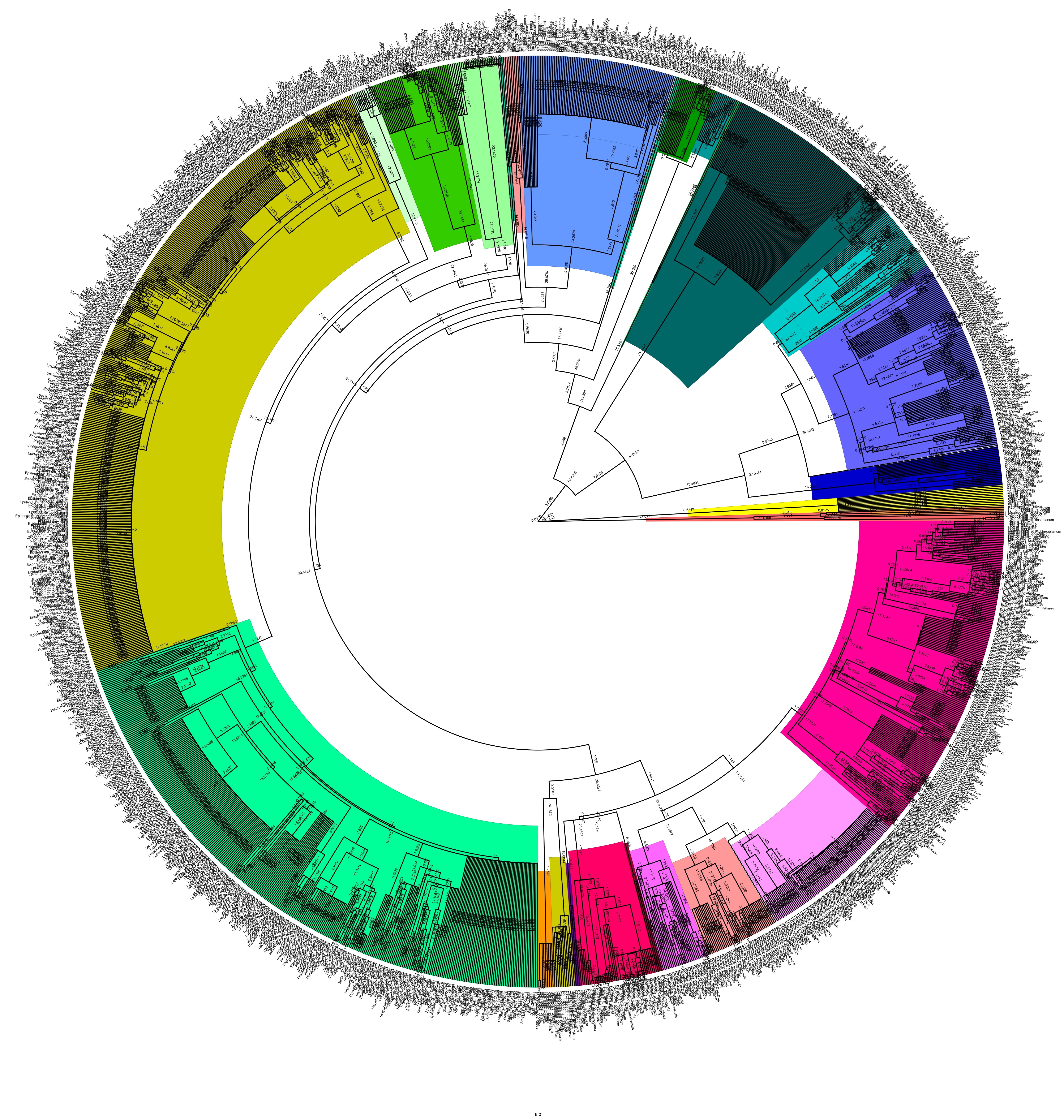

Supplement: Supplementary file 1 — Appendix A. Bibliographic sources for the life forms of the orchids of Megamexico. Appendix B. Database of the Orchidaceae of Megamexico including life form. Appendix C. Phylogenetic estimation of the 1732 orchids present in Megamexico using the method of Jin and Qian (2019). The numbers in the branches represent the age of the nodes. The grouping by color is done at the subtribe level. The subtribes in yellow belong to the Vanilloideae subfamily, in red to Cypripedioideae, in blues to Orchidoideae and in greens and pink to Epidendroideae. Appendix D. Corrected weighted endemism (CWE) of orchids of Megamexico. Appendix E. Grid cells with a high rate of endemism of the Orchidaceae of Megamexico. Appendix F. Frequency histogram of the ranges of species in km2 with distribution beyond Megamexico. [file mmc1.zip › Appendix C. Phylogeny.jpg]

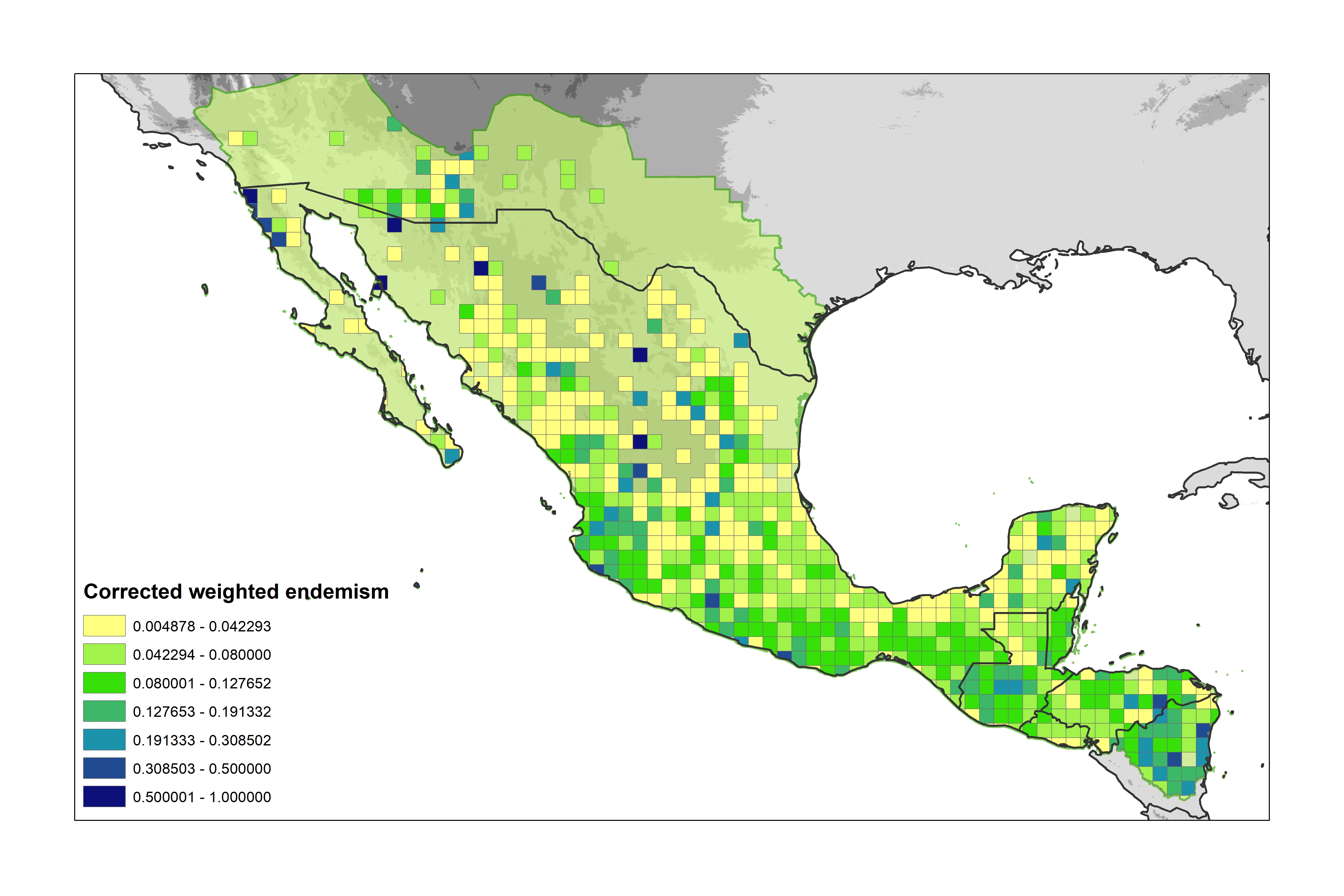

Supplement: Supplementary file 1 — Appendix A. Bibliographic sources for the life forms of the orchids of Megamexico. Appendix B. Database of the Orchidaceae of Megamexico including life form. Appendix C. Phylogenetic estimation of the 1732 orchids present in Megamexico using the method of Jin and Qian (2019). The numbers in the branches represent the age of the nodes. The grouping by color is done at the subtribe level. The subtribes in yellow belong to the Vanilloideae subfamily, in red to Cypripedioideae, in blues to Orchidoideae and in greens and pink to Epidendroideae. Appendix D. Corrected weighted endemism (CWE) of orchids of Megamexico. Appendix E. Grid cells with a high rate of endemism of the Orchidaceae of Megamexico. Appendix F. Frequency histogram of the ranges of species in km2 with distribution beyond Megamexico. [file mmc1.zip › Appendix D. CWE.jpg]

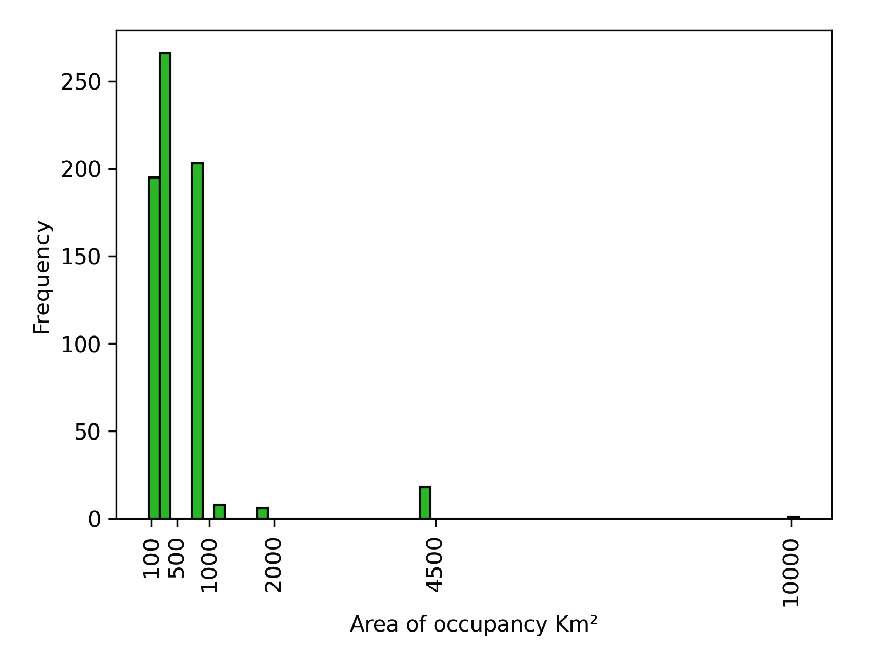

Supplement: Supplementary file 1 — Appendix A. Bibliographic sources for the life forms of the orchids of Megamexico. Appendix B. Database of the Orchidaceae of Megamexico including life form. Appendix C. Phylogenetic estimation of the 1732 orchids present in Megamexico using the method of Jin and Qian (2019). The numbers in the branches represent the age of the nodes. The grouping by color is done at the subtribe level. The subtribes in yellow belong to the Vanilloideae subfamily, in red to Cypripedioideae, in blues to Orchidoideae and in greens and pink to Epidendroideae. Appendix D. Corrected weighted endemism (CWE) of orchids of Megamexico. Appendix E. Grid cells with a high rate of endemism of the Orchidaceae of Megamexico. Appendix F. Frequency histogram of the ranges of species in km2 with distribution beyond Megamexico. [file mmc1.zip › Appendix F.jpg]
